# Bushen Tiaojing (II and III) Decoctions Activate MAPK14 and MAPK3/1 to Promote the Expression of Cumulus Expansion-Related Factors in Mice

**DOI:** 10.1155/2020/9283917

**Published:** 2020-02-18

**Authors:** Xiao Liang, Xue Tong, Hui-lan Du, Ming He, Yu Zhang, Yan-cang Duan

**Affiliations:** ^1^College of Integrated Traditional Chinese and Western Medicine, Hebei University of Chinese Medicine, Shijiazhuang, China; ^2^Collaborative Innovation Center of Integrated Chinese and Western Medicine on Reproductive Disease, Shijiazhuang, China; ^3^Hebei Key Laboratory of Integrative Medicine on Liver-Kidney Patterns, Shijiazhuang, China

## Abstract

**Background:**

Bushen Tiaojing Decoctions (BSTJ-II-D and BSTJ-III-D) are used to assist pregnancy in clinical practice. In this study, we explored the ability of sequential administration of BSTJ-II-D and BSTJ-III-D to promote cumulus cell (CC) expansion and its underlying mechanisms in controlled ovarian hyperstimulation (COH) mice.

**Methods:**

Kunming mice were randomly divided into three groups. The normal group was injected intraperitoneally with saline, and distilled water was administered orally by gavage. As the COH model, mice were injected with GnRHa, eCG, and hCG. Subsequently, the BSTJD group received BSTJ-II-D and BSTJ-III-D orally by gavage, while the control group received distilled water. We evaluated CC expansion and oocyte first polar body (PB1) extrusion under a stereomicroscope. Serum levels of follicle-stimulating hormone (FSH) were detected by radioimmunoassay. The expression of the CC expansion-related factors PTX3 and PTGS2 was detected by immunofluorescence, western blot, and quantitative real-time-polymerase chain reaction analyses (qRT-PCR). Expression of p-MAPK14, p-MAPK3/1, MAPK14, and MAPK3/1 was detected by western blot analysis.

**Results:**

Sequential administration of BSTJ-II-D and BSTJ-III-D promoted cumulus expansion and oocyte PB1 extrusion and upregulated PTX3 and PTGS2 expression at the mRNA and protein levels. Furthermore, the levels of p-MAPK14/MAPK14, p-MAPK3/1/MAPK3/1 proteins, and serum FSH in the BSTJD group were higher than those in the normal and control groups.

**Conclusions:**

Sequential administration of BSTJ-II-D and BSTJ-III-D promotes cumulus expansion and oocyte maturation in COH mice by increasing FSH expression and activating the MAPK14 and MAPK3/1 signalling pathways, thereby increasing expression of PTX3 and PTGS2.

## 1. Introduction

In recent years, the incidence of female infertility has increased rapidly in China. In vitro fertilization and embryo transfer (IVF-ET) technology is commonly used to treat this condition, and oocyte maturation is a key factor affecting the success of this method. Although the development of IVF-ET has contributed significantly to the treatment of infertility, the pregnancy and delivery rates of IVF-ET cycles remain relatively low [1]. Traditional Chinese Medicine (TCM) has played an important role in improving the success rate of IVF-ET [2]. According to the fundamental theory of TCM, Kidney is the congenial foundation, storing essence, and dominating development, growth, and reproduction. Sufficient kidney “Qi,” which is the basis for gestation, can be subdivided into “yin” and “yang.” Kidney yin nourishes follicles as the basis of follicular development, and the warming and promoting effects of kidney yang are fundamental to ovulation. Deficiency in kidney yin or yang can lead to essence and blood lacking source and thoroughfare-conception meridian disorder, and cause gynecological disorders such as menstrual dysfunction and infertility [3]. The kidney-tonifying method is commonly used to promote follicular development and maturation in clinical practice and has a remarkable curative effect [4, 5].

Bushen Tiaojing II Decoction (BSTJ-II-D) and Bushen Tiaojing III Decoction (BSTJ-III-D) are used to regulate menstruation and assist pregnancy in clinical practice. BSTJ-II-D is used in the postmenopause follicular phase, nourishing kidney yin, replenishing essence and blood, and balancing the thoroughfare and conception vessels to promote follicular development. BSTJ-III-D is used in the premenopausal luteal phase, warming kidney yang, regulating Qi and blood in thoroughfare and conception vessels, and promoting the transformation of extreme yin into yang during ovulation. Previous trials have shown that sequential use of BSTJ-II-D and BSTJ-III-D can increase the number of oocytes produced in ovulation cycles in a mouse model of controlled ovarian hyperstimulation (COH) [6]. However, the molecular mechanism by which BSTJ-II-D and BSTJ-III-D promote follicular development and maturation remains to be clarified.

Numerous factors, including oocyte maturation disorder, contribute to IVF-ET. Bidirectional communication between cumulus cells (CCs) and the oocyte plays a key role in oocyte development [7]. Cumulus expansion is closely related to gap junction integrity, which affects the exchange of substances between CCs and oocytes, as well as the recovery of oocyte meiosis [8]. Cumulus expansion can also be used as a predictive indicator of in vitro fertilization outcomes [9–11]. Therefore, cumulus expansion is an important parameter affecting the maturity of oocytes [12].

Cumulus expansion is the result of changes in the extracellular matrix (ECM). Pentraxin 3 (PTX3) acts as a central “node” in the formation of cumulus ECM by binding to tumor necrosis factor alpha-inducible protein 6 (TNFAIP6) and serum protein alpha-trypsin inhibitor (I*α*I) [13], which stabilizes the ECM [14]. Prostaglandin synthase 2 (PTGS2) is also necessary for cumulus expansion [15], and increasing expression can improve oocyte development [16, 17]. Cumulus expansion requires secretion of cumulus expansion-enabling factors (CEEFs) by the oocyte and stimulation by epidermal growth factor (EGF) or follicle-stimulating hormone (FSH). This combined stimulation results in activation of the MAPK3/1 and MAPK14 signaling pathways and increases PTGS2, TNFAIP6, and PTX3 mRNA levels, all of which are required for cumulus expansion [18, 19].

The grade of cumulus expansion can be classified according to the method described by Vanderhyden [20]. In mice, cumulus expansion is observed at 6 h after hCG injection, and the cumulus is completely expanded by 9 h after hCG injection [21]. At 12 h after hCG injection, the first polar body extrusion was observed with complete expansion of the cumulus [22]. Therefore, we chose 6 h, 9 h, and 12 h after hCG injection as the time points for our investigation of the ability of sequential administration of BSTJ-II-D and BSTJ-III-D to promote cumulus expansion and the underlying mechanism.

## 2. Materials and Methods

### 2.1. Animals

In total, 120 female Kunming mice (aged 8-9 weeks and weighing 25–30 g) (no. SCXK 2016-0011) were purchased from Beijing Vital River Laboratory Animal Technology Co., Ltd. (Beijing, China), The animal protocol was approved by the Ethics Committee of the Institute of Hebei University of Chinese Medicine (DWLL2018001, China) and in accordance with the Guide for the Care and Use of Laboratory Animals (National Academy of Sciences, copyright 1996).

### 2.2. Experimental Drugs

The BSTJ-II-D was composed of Shudihuang (*Radix Rehmanniae Preparata*) 15 g, Danggui (*Radix Angelicae Sinensis*) 9 g, Gouqizi (*Tructus Lycii*) 12 g, Nvzhenzi (*Fructus Ligustri Lucidi*) 9 g, Ziheche (*Placenta Hominis*) 3 g, Baishao (*Radix Paeoniae Alba*) 9 g, and Shanzhuyu (*Fructus Corni*) 15 g. The BSTJ-III-D was composed of Shudihuang (*Radix Rehmanniae Preparata*) 15 g, Xianmao (*Rhizoma Curculigins*) 12 g, Yinyanghuo (*Herba Epimedii*) 12 g, Danshen (*Radix Salviae Miltiorrhizae*) 10 g, Zishiying (*Fluoritum*) 10 g, Fupenzi (*Fructus Rubi*) 10 g, Roucongrong (*Herba Cistanches*) 10 g, Zhiqiao (*Fructus Aurantii*) 10 g, Danggui (*Radix Angelicae Sinensis*) 10 g, and Tusizi (*Semen Cuscutae*) 12 g. In this study, BSTJ-II-D was used during follicular development (i.e., 9 days before eCG injection) to nourish kidney yin, replenish essence and blood, and balance the thoroughfare and conception vessels to promote follicular development. BSTJ-III-D was used when ovulation was about to occur (i.e., 1 day before and after hCG injection) to warm kidney yang, regulate Qi and blood in the thoroughfare and conception vessels, and promote the transformation of extreme yin transforming into yang during ovulation. These herbals were purchased from Shijiazhuang Lerentang Pharmaceutical Co., Ltd. The Chinese herbal medicines were identified and decocted in water. BSTJ-II-D and BSTJ-III-D contained 1.05 g/ml and 1.43 g/ml of the drugs, respectively, and after sterilization with UV light for 1 h were stored at 4°C. GnRHa was purchased from Beijing Solarbio Technology Co., Ltd. (Beijing, China), and eCG and hCG were purchased from Hangzhou Animal and Drug Factory (Hangzhou, China).

### 2.3. Generation of the COH Mouse Model and Grouping

At 9 : 00 daily, female Kunming mice were observed for the occurrence of two estrus cycles induced by vaginal detachment. The mice with regular estrus cycles were randomly divided into the normal, control, and BSTJD groups (*n* = 40 mice per group). The COH model was established according to a previously described method [23]. Briefly, mice with regular estrus cycles were injected intraperitoneally with GnRHα 40 *μ*g/100 g daily at 9 : 00 in the late estrus period for 9 consecutive days. On day 9, eCG 40 IU/100 g was administered simultaneously, followed by hCG 100 IU/100 g 48 h later. Mice in the normal group were injected with an equal volume of 0.9% saline. While establishing the COH model, mice in the BSTJD group received oral BSTJ-II-D 18.70 g/kg daily by gavage on days 1 to 9 followed by BSTJ-III-D 28.57 g/kg on days 10 and 11. Mice in the normal and control group were given the same volume of distilled water.

### 2.4. Acquisition of Cumulus-Oocyte Complexes (COCs)

In the normal group, mice were sacrificed by cervical dislocation at 6 h, 9 h, and 12 h after the estrus was observed, whereas mice in the control and BSTJF groups were sacrificed at 6 h, 9 h, and 12 h after hCG injection. The ovaries were quickly removed and placed in Eagle's minimum essential medium-alpha modification (*α*-MEM). Under a stereomicroscope, the ovary was gently ruptured using a sterile 1 mL syringe needle, and the larger follicles were detached. Free COCs were removed, transferred to a 1.5 mL Eppendorf tube with a pipette and stored to −80°C.

### 2.5. Testing Indexes and Methods

#### 2.5.1. Grading of Cumulus Expansion

COCs were counted and cumulus expansion was graded according to the following criteria [20]: Grade 0 (G0), no expansion; Grade 1 (G1), smallest degree of expansion, with radial distribution of the COCs; Grade 2 (G2), slight expansion of the outer CC layer; Grade 3 (G3), all CCs have expanded except those in the radiating canopy CCs that are close to the oocyte; and Grade 4 (G4), full expansion of CC layers.

#### 2.5.2. Evaluation of Nuclear Maturation

We obtained COCs at 12 h after hCG injection to study the effect of BSTJD on nuclear maturation of oocytes. After assessment of cumulus expansion, we removed CC by gentle mechanical pipetting of COC using a 20 *μ*L pipette. Nuclear maturation was recorded as the extrusion of the first polar body (PB1) from the oocyte. The number and proportion of PB1 oocytes were calculated.

#### 2.5.3. Measurement of Serum FSH Levels by Radioimmunoassay

Mice in the normal group were anesthetized with ether at 6 h, 9 h, and 12 h after the estrus was observed, and mice in the control and BSTJF groups were anesthetized at 6 h, 9 h, and 12 h after the hCG injection. Blood samples were obtained by decapitation and centrifuged at 5,000 rpm for 5 min at 4°C to separate the serum, which was stored at −80°C. Serum FSH levels were then determined using a FSH radioimmunoassay kit (Jiuding Medical Bioengineering Co., Ltd, Tianjin, China) according to the manufacturer's instructions.

#### 2.5.4. Quantitative Real-Time Polymerase Chain Reaction (qRT-PCR)

The PTX3 and PTGS2 mRNA levels in COCs were measured by quantitative real-time polymerase chain reaction (qRT-PCR). Total RNA was extracted from COCs using the Total RNA Kit II (Omega Bio-Tek, Norcross, GA, USA) according to the manufacturer's instructions. The purity of the RNA was determined using a Nanodrop 2000C spectrophotometer (Thermo Fisher Scientific, Waltham, MA, USA) to determine the OD260/OD280 of the extracted RNA, with values in the range of 1.8–2.0 considered to be of high purity. Using RNA as the template, cDNA was generated by reverse transcription using a two-step reverse transcription kit (Vazyme Biotech Co., Ltd, Nanjing, China) according to the manufacturer's instructions. Following removal of the genomic DNA, cDNA was generated in a 20 *μ*L reverse transcription reaction system real-time fluorescent quantitative PCR reaction on an Applied Biosystems 7500 Fast Real-Time PCR system (Thermo Fisher Scientific). The PCR thermal cycle parameters were as follows: predenaturation at 95°C for 5 min followed by 40 cycles of 95°C for 10 min and 60°C for 30 s. The following primers were designed and synthesized by Sangon Biotech (Shanghai, China) Co., Ltd.: GAPDH (internal reference), Forward: 5′-GGTTGTCTCCTGCGACTTCA-3′, Reverse: 3′-TGGTCCAGGGTTTCTTACTCC-5′ (183 bp); PTX3, Forward: 5′-GCTTCACTCCTGCCTCACACTATC-3′, Reverse: 3′-ATCTGCGAGTTCTCCAGCATGATG-5′ (307 bp); PTGS2: Forward: 5′-TGGTCTGGTGCCTGGTCTGATG-3′, Reverse: 3′-GCGGTTCTGATACTGGAACTGCTG-5′ (253 bp). After the amplification, the CT values for each sample were determined. Using the first sample of the normal group as the standard (assigned an arbitrary value of 1), the relative quantitative value (RQ value) of the target gene expression was calculated according to the formula RQ = 2^−ΔΔCt^, and the RQ value was used for statistical analysis.

#### 2.5.5. Immunofluorescence

COCs were fixed with 4% paraformaldehyde and then incubated with 0.5% Triton X-100 phosphate buffer saline (PBS) for 15 min at room temperature to increase cell permeability. After incubation with 10% goat serum to block nonspecific antigen binding, the COCs were incubated with anti-PTX3 antibody (PA5-38595; Thermo Fisher Scientific; 1 : 100; Waltham, MA, USA) and anti-PTGS2 antibody (ab90345; Abcam; 1 : 100; Cambridge; UK) overnight at 4°C. The next day, the COCs were incubated with a fluorescently labeled secondary detection antibody (SA00008-2; Proteintech; 1 : 200; Chicago, IL, USA) in a humid chamber at 37°C for 1 h in the dark. The COCs were counterstained with 4′,6-diamidino-2-phenylindole (DAPI) prior to analysis under a fluorescence microscope (Thermo Fisher Scientific).

#### 2.5.6. Western Blot Analysis

Total protein was extracted from COCs with RIPA lysis buffer (Beijing Solarbio Science and Technology Co., Ltd., Beijing, China) on ice. The protein extraction solution was adjusted to a final concentration of 1% PMSF (Beijing Solarbio Science and Technology Co., Ltd.) and 1% phosphatase inhibitors (MCE, NJ, USA). The protein concentration was determined using the BCA Protein Assay Kit (Beijing Solarbio Science and Technology Co., Ltd.) according to the manufacturer's instructions. Proteins were then stored at −80°C prior to analysis. Protein samples (20 *µ*g) were mixed with 5× SDS buffer (Beijing Solarbio Science and Technology Co., Ltd.) and heated at 100°C for 8 min to denature the protein. The proteins were separated by sodium dodecyl sulfate-polyacrylamide gel electrophoresis (SDS-PAGE) before being transferred electrophoretically onto a polyvinylidene fluoride (PVDF) membrane. After blocking with 5% nonfat milk for 2 h at room temperature and washing with Tris buffered saline-Tween-20 (TBST), the membrane was incubated overnight at 4°C with anti-PTX3 antibody (1 : 1,000; PA5-38595; Thermo Fisher Scientific, Waltham, MA, USA), anti-PTGS2 antibody (1 : 2,000; ab90345; Abcam, Cambridge; UK), anti-MAPK14 antibody (1 : 2,000; ab32142; Abcam), anti-MAPK3/1 antibody (1 : 1,000; ab17942; Abcam), anti-p-MAPK14 antibody (1 : 1,000; ab4822; Abcam), anti-p-MAPK1/2 antibody (1 : 1,000; ab50011; Abcam), and anti-GAPDH (internal reference) antibody (1 : 1,000; 10494-I-AP, Proteintech; Chicago, IL, USA) followed by incubation with HRP-conjugated Affinipure goat anti-rabbit IgG (1 : 1,000; SA00001-2; Proteintech) for 2 h at room temperature. The bands were visualized using a chemiluminescence imaging system (GE, Boston, MA, USA) with Immobilon® Western chemiluminescent HRP substrate (EMD Millipore, Inc., Billerica, MA, USA). The protein bands were analyzed using the ImageQuant TL Image Analysis System (GE Amersham Biosciences, Boston, MA, USA). GAPDH expression was used as the internal reference.

### 2.6. Statistical Analysis

All data were processed with SPSS 21.0 (IBM SPSS Statistics for Windows, IBM Corp., Armonk, NY, USA) and expressed as the mean ± standard deviation (SD). Grades of cumulus expansion grading at three times were compared using the repeated measurements analysis of variance (RM-ANOVA). Variables of interest were compared between groups by one-way analysis of variance and followed by least squares difference tests (LSD) or Dunnett's T3 tests. *P* values less than 0.05 were considered to indicate statistical significance.

## 3. Results

### 3.1. Grading of Cumulus Expansion in COCs

To explore the ability of sequential administration of BSTJ-II-D and BSTJ-III-D to promote cumulus expansion, we examined the number and grade of cumulus expansion and calculated the ratio of cumulus expansion at different grades ([Supplementary-material supplementary-material-1]). RM-ANOVA showed that time had a statistically significant impact on the cumulus expansion grading, indicating that the proportion of high-grade cumulus expansion increased with time (*P* < 0.05). In terms of the cumulus expansion grading in different groups, the ratio of high-grade cumulus expansion of COCs in the BSTJD group was higher than that in the normal and control groups, while the ratio of low-grade cumulus expansion of COCs in the BSTJD group was lower (*P* < 0.05).

### 3.2. Evaluation of Nuclear Maturation in Oocytes at 12 h after hCG Injection

**Figure 1:**
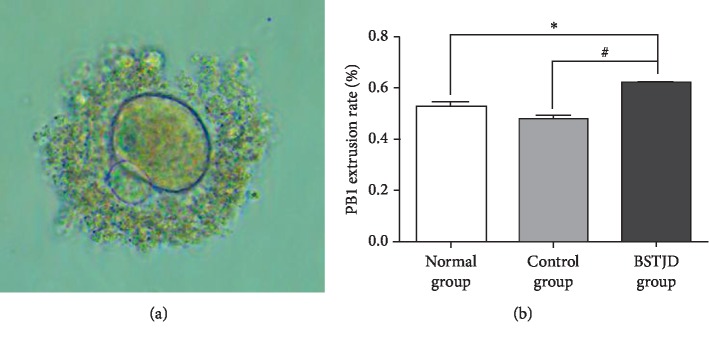
Evaluation of nuclear maturation in oocytes in the three groups at 12 h after hCG injection. (a) PB1 extrusion. Magnification: 200×. (b) PB1 extrusion rate in the three groups at 12 h after hCG injection. Data represent the mean ± SD, *n* = 9; ^*∗*^*P* < 0.05 compared with the normal group; ^#^*P* < 0.05 compared with the control group.

In our study, nuclear maturation was defined as the extrusion of the first polar body (PB1) from the oocyte. The number and proportion of PB1 oocytes are shown in Figure 1. Compared with the normal group, there was no significant difference in the ratio of PB1 oocytes in the control group at 12 h after saline or hCG injection (*P* > 0.05), while the ratio of PB1 oocytes in the BSTJD group was significantly increased at 12 h after saline or hCG injection (*P* < 0.05).

### 3.3. Serum FSH Levels at Different Times after hCG Injection

To further study the mechanism by which BSTJD promotes cumulus expansion, we evaluated serum FSH levels in each group by radioimmunoassay (Table 1). At 6 h, 9 h, and 12 h after saline or hCG injection, there were no significant differences in serum FSH levels between the normal and control groups (*P* > 0.05), while serum FSH levels in the BSTJD group were significantly increased at 6 h and 12 h after saline or hCG injection (*P* < 0.05).

### 3.4. Expression and Localization of Cumulus Expansion-Related Proteins in COCs at Different Times after hCG Injection

**Figure 2:**
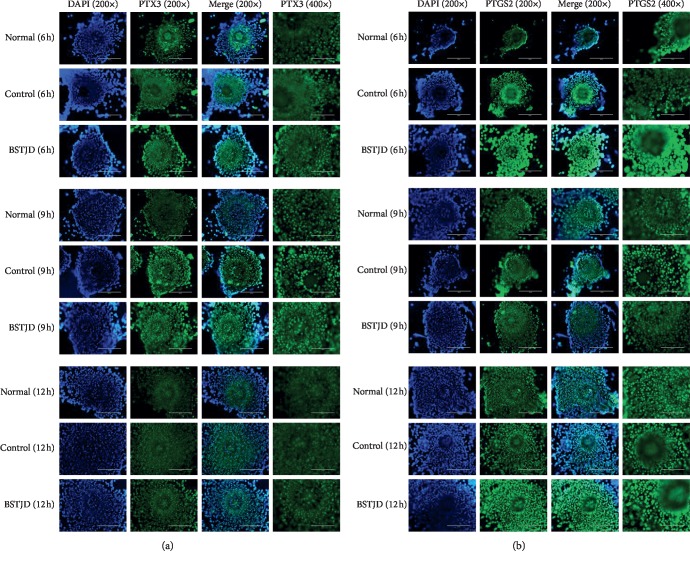
Immunofluorescence staining. (a) Expression of PTX3 protein in COCs in the three groups at different times. Magnification: images in columns 1, 2, and 3, 200×, scale = 200 *μ*m. Images in column 4, 400×, scale = 100 *μ*m. (b) Expression of PTGS2 protein in COCs in the three groups at different times. Magnification: images in columns 1, 2, and 3, 200×, scale = 200 *μ*m. Images in column 4, 400×, scale = 100 *μ*m.

We speculated that the role of BSTJD in promoting cumulus expansion was related to the expression of cumulus expansion-related factors. Therefore, we conducted immunofluorescence analysis of the expression and localization of cumulus expansion-related proteins in the COCs of three groups of mice at 6 h, 9 h, and 12 h after saline or hCG injection (Figure 2). Both PTX3 and PTGS2 proteins were expressed in CC and oocytes, and the fluorescence intensity of PTX3 and PTGS2 protein in the BSTJD group was greater than that in the normal and control groups.

### 3.5. Expression of PTX3 and PTGS2 Proteins in COCs at Different Times after hCG Injection

**Figure 3:**
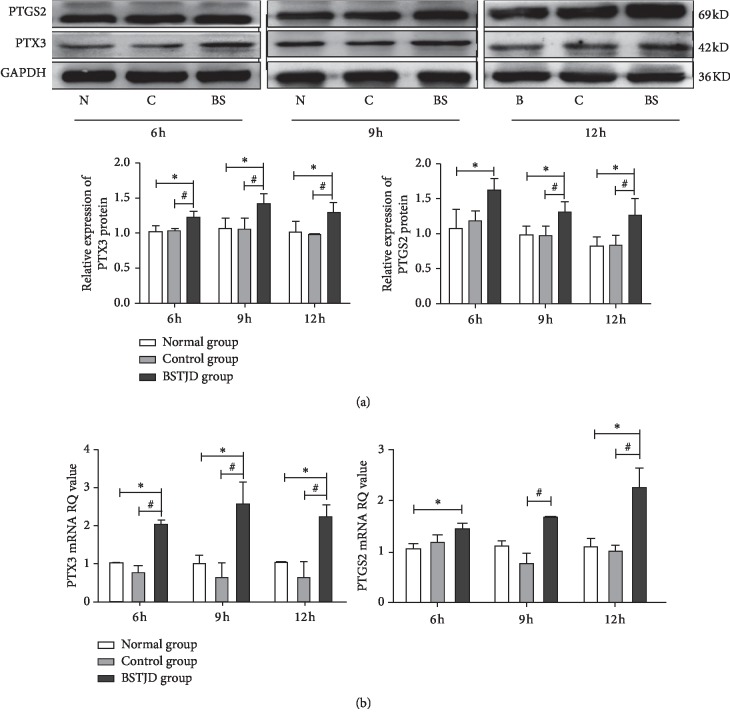
Western blot and qRT-PCR analyses. (a) Western blot analysis of the expression of PTX3 and PTGS2 proteins in COCs in the three groups at different times. N: normal group; C: control group; BS: BSTJD group. (b) qRT-PCR analysis of the expression of PTX3 and PTGS2 mRNA in COCs in the three groups at different times. Data represent the mean ± SD, *n* = 3; ^*∗*^*P* < 0.05 compared with the normal group; ^#^*P* < 0.05 compared with the control group.

Western blot analysis was then conducted to further confirm the ability of BSTJD to upregulate the expression of cumulus expansion-related proteins (Figure 3(a)). At 6 h, 9 h, and 12 h after saline or hCG injection, there were no significant differences in PTX3 and PTGS2 protein expression between the normal and control groups (*P* > 0.05), while PTX3 protein expression was significantly increased in the BSTJD group (*P* < 0.05). At 6 h after saline or hCG injection, PTGS2 protein expression was significantly increased in the BSTJD group compared with that in the normal group (*P* < 0.05). At 9 h and 12 h after saline or hCG injection, the expression of PTGS2 protein in the BSTJD group was significantly increased compared with that in the normal and control groups (*P* < 0.05).

### 3.6. Expression of PTX3 and PTGS2 mRNA in COCs at Different Times after hCG Injection

The effects of BSTJD on the expression of HAS2 and TNFAIP6 mRNA levels were analyzed by qRT-PCR (Figure 3(b)). There were no significant differences in the levels of PTX3 and PTGS2 mRNA between the normal and control groups (*P* > 0.05), while PTX3 mRNA levels were significantly increased in the BSTJD group (*P* < 0.05). At 6 h after saline or hCG injection, PTGS2 mRNA levels in the BSTJF group were significantly increased compared with those in the normal group (*P* < 0.05). At 9 h after hCG injection, PTGS2 mRNA levels in the BSTJF group were significantly increased compared with those in the control group (*P* < 0.05). At 12 h after saline or hCG injection, PTGS2 mRNA levels in the BSTJF group were significantly increased compared with those in the normal and control groups (*P* < 0.05).

### 3.7. Expression Levels of p-MAPK14/MAPK14, and p-MAPK3/1/MAPK3/1 Proteins in COCs at Different Times after hCG Injection

**Figure 4:**
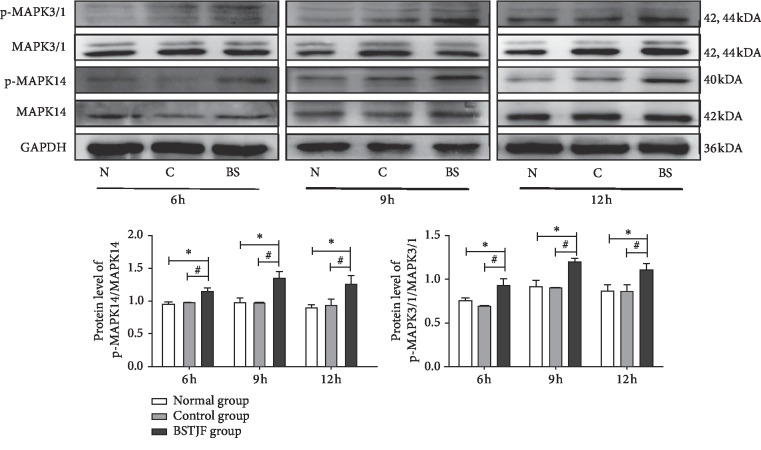
Western blot analysis of the expression levels of p-MAPK14/MAPK14 and p-MAPK3/1/MAPK3/1 proteins in COCs in the three groups at different times. Values are expressed as the Mean ± SD, *n* = 3; ^*∗*^*P* < 0.05 compared with the normal group; ^#^*P* < 0.05 compared with the control group.

After establishing the ability of BSTJD to promote the expression of cumulus expansion-related factors, we investigated the expression of the classical MAPK14 and MAPK3/1 signaling pathway-related proteins in COCs (Figure 4). At 6 h, 9 h, and 12 h after saline or hCG injection, there were no significant differences in the expression of p-MAPK14/MAPK14 and p-MAPK3/1/MAPK3/1 proteins between the normal and control groups (*P* > 0.05), while the expression of p-MAPK14/MAPK14 and p-MAPK3/1/MAPK3/1 proteins was significantly increased in the BSTJD group (*P* < 0.05).

## 4. Discussion

“Kidney stores essence and dominates reproduction” is the basic tenet of the role of TCM in human reproductive function. The kidney is the congenial foundation, playing a leading role in all biological activities. Kidney essence includes congenital essence and acquired essence, and it is the source of growth, development, and reproduction. Congenital essence stored in the kidney includes reproduction essence, and this essence can produce blood. The essence and blood are homologous and represent the material basis of menstruation and fetal pregnancy. Therefore, the kidney-tonifying method has been commonly used in gynecology for thousands of years, with positive effects. Previous studies have confirmed that the sequential administration of BSTJ-II-D and BSTJ-III-D can regulate the reproductive function of mice through multiple systems, multiple targets, and multiple links, increasing the number of follicles, improving follicle quality, and promoting follicular development and ovulation [24, 25]. This has also been confirmed by the results of modern pharmacological research. The efficacy of *Radix Rehmanniae Preparata* nourishing yin, nourishing blood, and replenishing essence is related to the effect of the medicine on the body's immune function, endocrine function, and its effects on hematopoietic and blood systems [26]. Drugs such as *Tructus Lycii, Placenta Hominis,* and *Rhizoma Curculigins* have pseudohormonal effects, which can promote ovarian secretion, protect the reproductive system, and improve reproductive capacity [27–29]. *Radix Angelicae Sinensis* can increase ovarian blood flow, improve microcirculation, and promote oocyte development and ovulation [30].

In the later stages of follicular development, granulosa cells differentiate into two functionally distinct populations: cells that surround the oocytes and maintain contact with the oocytes, called CCs, and cells located on the outer boundary of the follicles, called mural granulosa cells. Before ovulation, stimulated by a series of endogenous gonadotropin peaks, the tight junctions of COCs expand, a phenomenon known as cumulus expansion or mucification. Before ovulation, CCs regulate the level of cyclic adenosine monophosphate in oocytes to promote oocyte meiosis, changes in cumulus morphology and cumulus expansion to affect oocyte maturation. After ovulation, CCs affect sperm-oocyte binding and embryo development [31–33]. Therefore, cumulus expansion is a prerequisite for oocyte maturation and fertilization, and can also be used as a morphological indicator to predict oocyte quality [34, 35]. Promoting cumulus expansion has great significance for promoting oocyte maturation and increasing ovulation rates. Therefore, we investigated the ability of BSTJ-II-D and BSTJ-III-D to promote cumulus expansion.

In our study, a COH mouse model was established using GnRHa + eCG + hCG to simulate COH during IVF-ET. First, we evaluated the grades of cumulus expansion and PB1 extrusion from oocytes after the sequential administration of BSTJ-II-D and BSTJ-III-D in COH mice. The results indicated that the kidney-tonifying treatment promoted cumulus expansion and nuclear maturation of oocytes in mice. However, its specific mechanism needs further exploration.

Cumulus expansion refers to the expansion of CCs surrounding a mucin-like cell matrix in a process that is closely related to changes in the cumulus ECM. PTX3 is an essential structural component of the cumulus ECM. A stable ECM is not formed in PTX3-knockout mice, which affects oocyte quality and fertilization, leading to serious defects in female fertility [36, 37]. Prostaglandins (PGs) are important intrafollicular signaling molecules that play an important role in cumulus expansion, ECM formation, and ovulation [31]. PTGS2 catalyzes the production of PG precursors from membrane lipids. Female mice lacking either PTGS2 or the PGE (2) receptor EP2 are infertile, showing decreased ovulation, and abnormal cumulus expansion [16, 38]. FSH plays a key regulatory role in the antral and preovulatory follicular phase through multiple signal cascades [39]. The MAPK14 pathway is critical for FSH-induced epidermal growth factor (EGF) receptor transactivation. Selective knockdown of the MAPK14 gene in CCs prevents cumulus expansion and expression of certain genes, such as PTGS2 [40, 41]. MAPK3/1 activation in different species also plays a crucial role in cumulus expansion [42–44]. MAPK3/1 in CC is involved in transcriptional regulation and posttranscriptional events required for meiotic recovery and cumulus expansion [19]. Therefore, we hypothesized that the sequential administration of BSTJ-II-D and BSTJ-III-D would promote the expression of cumulus expansion-related factors by regulating FSH levels to activate MAPK14 and MAPK3/1.

We evaluated the effect of BSTJ-II-D ang BSTJ-III-D on cumulus expansion in mice by detecting the expression and localization of proteins necessary for cumulus expansion, such as PTX3 and PTGS2, by western blot and immunofluorescence analyses. Our results showed that BSTJ-II-D and BSTJ-III-D increased the expression of these two proteins at the mRNA and protein levels, which suggested that the sequential administration of BSTJ-II-D and BSTJ-III-D promotes cumulus expansion by enhancing the expression of the cumulus expansion-related factors. In addition, we detected the p-MAPK/MAPK14 and p-MAPK3/1/MAPK3/1 expression in COCs of mice by western blot and serum FSH of mice by radioimmunoassay. Our experiments confirmed the increase in p-MAPK/MAPK14 and p-MAPK3/1/MAPK3/1 expression and serum FSH level in the BSTJD group, elucidating the possible mechanism by which the sequential administration of BSTJ-II-D and BSTJ-III-D promotes cumulus expansion. These results confirmed our hypothesis.

## 5. Conclusions

In conclusion, our results indicate that the sequential administration of BSTJ-II-D and BSTJ-III-D promotes cumulus expansion and oocyte maturation in COH mice by increasing FSH expression and activating the MAPK14 and MAPK3/1 signaling pathways, thereby increasing expression of PTX3 and PTGS2. However, the ability of kidney-tonifying method to affect oocyte maturation by promoting cumulus expansion remains to be fully elucidated.

## Figures and Tables

**Table 1:** Serum FSH level in the three groups at different times ($\bar{x}$ ± SD).

Group	*n*	6 h FSH (mIU/mL)	9 h FSH (mIU/mL)	12 h FSH (mIU/mL)
Normal group	9	7.36 ± 0.60	7.89 ± 0.83	5.56 ± 0.71
Control group	9	7.04 ± 0.61	7.17 ± 1.01	5.72 ± 0.62
BSTJF group	9	10.80 ± 0.38^*∗*#^	10.41 ± 0.86^#^	8.21 ± 0.60^*∗*#^

^*∗*^ *P* < 0.05 compared with the normal group; ^#^*P* < 0.05 compared with the control group

## Data Availability

The data used to support the findings of this study are available from the corresponding author upon request.

## Supplementary Materials

Supplementary Materials Supplementary Figure 1: Grading of cumulus expansion in COCs in the three groups 22 at different times. (a) Images from left to right correspond to cumulus expansion grades 0–4. Magnification: images 1-3, 400×; images 4-5, 200×. (b) Grading of cumulus expansion in COCs in the three groups at 6 h after hCG injection. (c)Grading of cumulus expansion in COCs in the three groups at 9 h after hCG injection. (d) Grading of cumulus expansion in COCs in the three groups at 12 h after hCG injection. Data represent the mean ± SD, *n* = 9; ^*∗*^*P* < 0.05 compared with the normal group; ^#^*P* < 0.05 compared with the control group. Supplementary Figure 2: Immunofluorescence staining. (a) Expression of PTX3 protein in COCs in the vascular endothelial cell. Magnification: 200×, scale = 200 *μ*m. (b) Expression of PTGS2 protein in COCs in the vascular endothelial cell. Magnification: 200×, scale = 200 *μ*m.
