# Supplementary material for: Bushen Tiaojing (II and III) Decoctions Activate MAPK14 and MAPK3/1 to Promote the Expression of Cumulus Expansion-Related Factors in Mice
Source: Evid Based Complement Alternat Med. 2020 Feb 18;2020:9283917. doi: 10.1155/2020/9283917 (PMC7049407; doi:10.1155/2020/9283917)
Supplement: Supplementary Materials — Supplementary Figure 1: Grading of cumulus expansion in COCs in the three groups 22 at different times. (a) Images from left to right correspond to cumulus expansion grades 0–4. Magnification: images 1-3, 400×; images 4-5, 200×. (b) Grading of cumulus expansion in COCs in the three groups at 6 h after hCG injection. (c)Grading of cumulus expansion in COCs in the three groups at 9 h after hCG injection. (d) Grading of cumulus expansion in COCs in the three groups at 12 h after hCG injection. Data represent the mean ± SD, n = 9; ∗P < 0.05 compared with the normal group; #P < 0.05 compared with the control group. Supplementary Figure 2: Immunofluorescence staining. (a) Expression of PTX3 protein in COCs in the vascular endothelial cell. Magnification: 200×, scale = 200 μm. (b) Expression of PTGS2 protein in COCs in the vascular endothelial cell. Magnification: 200×, scale = 200 μm. [file 9283917.f1.pdf]

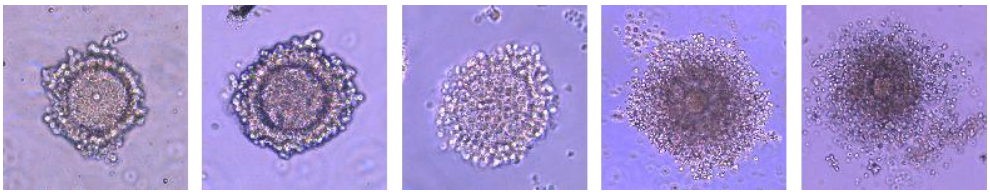

(a)

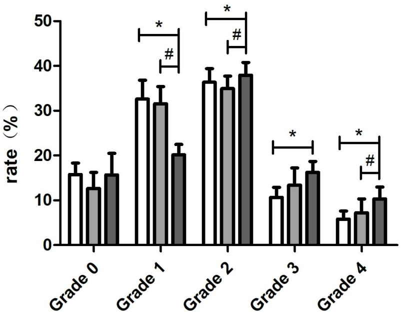

(b)

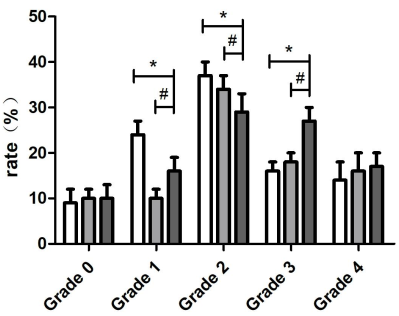

(c)

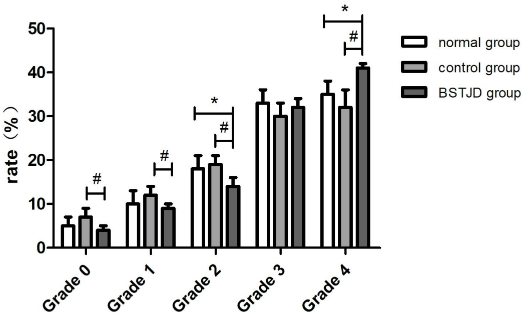

(d)

FIGURE S1: Grading of cumulus expansion in COCs in the three groups at different times. (a): Images from left to right correspond to cumulus expansion grades 0-4. Magnification: Images 1–3, 400×; Images 4–5, 200×. (b): Grading of cumulus expansion in COCs in the three groups at 6 h after hCG injection. (c): Grading of cumulus expansion in COCs in the three groups at 9 h after hCG injection. (d): Grading of cumulus expansion in COCs in the three groups at 12 h after hCG injection. Data represent the mean ± SD, n = 9; * $P < 0.05$ compared with the normal group; # $P < 0.05$ compared with the control group.

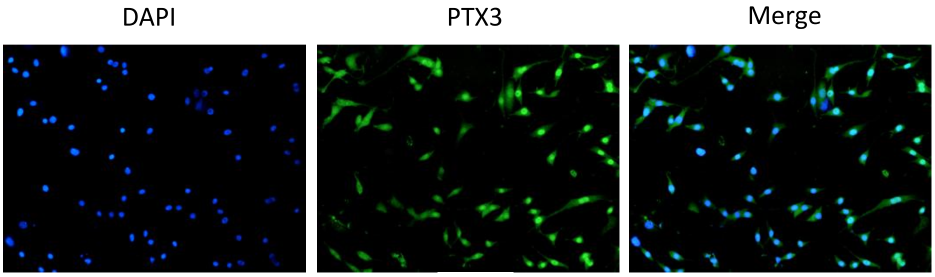

(a)

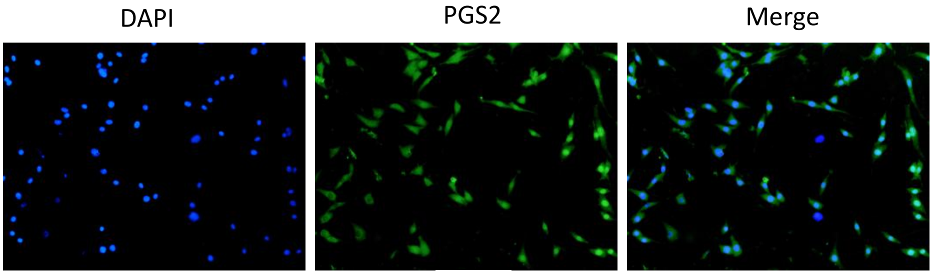

(b)

FIGURE S2: Immunofluorescence staining. (a) Expression of PTX3 protein in COCs in the vascular endothelial cell. Magnification: 200×, scale = 200 μm. (b) Expression of PTGS2 protein in COCs in the vascular endothelial cell. Magnification: 200×, scale = 200 μm.
